# Symptoms during initiation of a ketogenic diet: a scoping review of occurrence rates, mechanisms and relief strategies

**DOI:** 10.3389/fnut.2025.1538266

**Published:** 2025-03-26

**Authors:** Oddbjørn Skartun, Callum Rhys Smith, Johnny Laupsa-Borge, Simon Nitter Dankel

**Affiliations:** Department of Clinical Science, University of Bergen, Bergen, Norway

**Keywords:** ketogenic diet, ketogenesis, keto-induction, keto flu, symptoms, side-effects

## Abstract

**Background:**

Evidence for the clinical utility of ketogenic diets (KD) is mounting. The transition to a KD (keto-induction) can however trigger unpleasant transient symptoms (≪keto-flu≫) which may deter continued adherence. Knowledge of strategies that mitigate symptoms during keto-induction may facilitate adoption of a KD.

**Aim:**

We aimed to perform a scoping review of the available scientific literature with regards to symptom occurrence rates, possible mechanisms and proposed interventions for symptom relief during keto-induction.

**Methods:**

Embase, Medline and Web of Science electronic databases were searched systematically using terms associated with the KD and keto-induction in conjunction with terms capturing adverse effects. In addition, additional relevant studies were retrieved from the identified articles' references.

**Results:**

The available literature on keto-induction symptoms is highly heterogenous, but common transient symptoms are reported across multiple populations, including descriptions of “keto-flu,” nausea, emesis, reduced appetite, hypoglycaemia, acidosis, increased risk of kidney stones, altered liver biochemistry, and skin rash. Mechanisms have been proposed based on general insights into physiology, but few have been empirically tested. However, approaches to reduce symptoms of keto-initiation are reported, including avoidance of the traditionally used fasted initiation and supplementation of medium-chain triglycerides (MCT) and ketone salts. There is a physiological rationale for supplementation with electrolytes and ketone esters, but a lack of clinical studies documenting their effect.

**Conclusion:**

Several transient symptoms have been associated with keto-induction, although a limited number of studies have directly examined them, or the mechanisms and possible interventions for symptom alleviation. Further research is warranted to close knowledge gaps highlighted in this review.

## 1 Introduction

A ketogenic diet (KD) is characterized by a very low intake of carbohydrates, moderate protein intake, and typically a higher intake of fats to maintain a balanced total energy intake (1). The low availability of dietary carbohydrate maintains a predominant oxidation of fatty acids rather than glucose, mimicking a starvation state that involves increased ketogenesis primarily in the liver. Ketogenesis involves production of the ketone bodies β-hydroxybutyrate (βHB), acetoacetate (AcAc) and acetone, which serve as an alternative fuel substrate for the body's cells. For example, the blood brain barrier is permeable to ketone bodies and they are readily utilized to cover up to 70% of the brain's energy requirements under conditions of reduced glucose availability (2–4). Nutritional ketosis (NK) is a state we have relied on evolutionarily in times of low dietary carbohydrate availability, and should not be confused with the pathological and dangerous state of ketoacidosis. Rather than a normal physiological adaptation, ketoacidosis is the result of a metabolic dysregulation that for example occurs in alcoholism and uncontrolled type 1 diabetes (5). NK is typically defined by a circulating blood βHB concentration above 0.5 mmol/L (6), and can yield βHB concentrations above 2 mmol/L when maintained over time (7).

**Figure 1:**
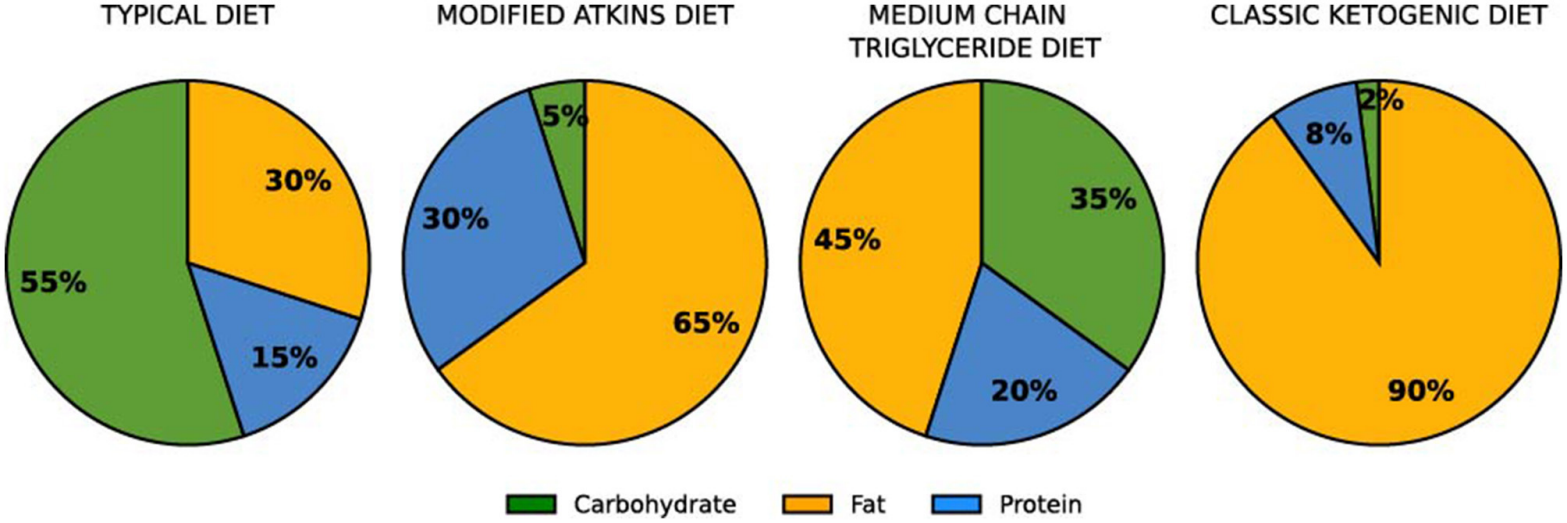
Macronutrient ratios used in some standard diet formulations [calculated by mass, not energy percentage of total energy; (6)].

KDs are being intensely studied as options for the treatment of disorders that involve metabolic changes, including obesity, type 2 diabetes, cardiovascular disease, cancer and dementia (8–13). For more than a century, KDs have been used for the treatment of intractable epilepsy in both children and adults (14–16). A classical KD is typically defined by a macronutrient mass ratio of 4:1 of fat to non-fat, and 50 g of carbohydrate per day is commonly agreed to be a cut-off point for entry into ketosis (17, 18) (Figure 1). Ongoing research shows promising neuroprotective effects of a KD with implications also for Alzheimer's disease, Parkinson's disease, multiple sclerosis, Huntington's, autism spectrum disorder, stroke, motor neuron disease, malignant glioma, migraine headache and traumatic brain injury (10, 17, 19). A significant interest in KD is centered around the direct metabolic effects, such as for achieving weight loss and improved insulin sensitivity in obesity, type 2 diabetes and polycystic ovarian syndrome (20), and for fine-tuning athletic performance by pushing fuel substrate utilization in the direction of fats (6). On a KD, athletes can obtain mean levels of maximal fat oxidation of over 1.5 g min^−1^ which is around 200–250% of the typical levels on a high-carbohydrate diet (21).

As well as providing an alternative fuel source for the brain, ketone bodies are thought to regulate the nervous system. Firstly, ketone bodies act as ligands for several G protein coupled receptors, thus orchestrating cellular responses and acting as signaling molecules in pathways related to lipid metabolism and cellular growth (22). Additionally, ketone bodies have been found to play an active role in the regulation of neuronal excitability by decreasing neuronal firing. Mitochondrial ketolysis increases the synthesis and accumulation of GABA, an inhibitory neurotransmitter in GABAergic neurons (10). It also reduces the release of glutamate, thereby lowering extracellular levels of this excitatory neurotransmitter (17). During ketosis, intracellular glycolytic activity is diminished, and hence cytosolic ATP levels are lowered (10). This is thought to play an important role in decreasing neuronal excitability thanks to the opening of potassium-sensitive ATP channels, leading to hyperpolarization of the neuronal membrane (10). Furthermore, ketone bodies can exert neuroprotection via other mechanisms including upregulation of autophagy in proteostasis, stimulation of cerebral blood flow and angiogenesis, epigenetic regulatory activity and an array of anti-inflammatory effects (10, 22). Some of these properties are also of great interest outside the field of neurology. For example, ketosis induced using medium-chain triglycerides (MCT) has recently shown promise for easing rheumatoid arthritis symptoms (23).

Clinical applicability of KDs is also reported for both autosomal dominant polycystic kidney disease (ADPKD) and cancer. In ADPKD, cyst linings are thought to be metabolically inflexible and reliant on glucose, thus the lowering of available glucose via a KD provides a potentially promising means of combating cyst development (24). In cancer research there has been a resurgence of interest in targeting metabolic mediators of the Warburg effect, whereby cancer cells upregulate anaerobic glycolysis even in the presence of sufficient oxygen. The KD as a means of restricting tumor glucose supply was first investigated in 1941 (9). More recent research interest has been centered around ketone bodies themselves playing a role in epigenetic alterations in tumor cells as well as the effect of lowered insulin levels (resulting from reduced glucose availability) on oncogenic signaling pathways (9). Consistent with a potential cancer-protecting and anti-aging effect, a systematic review of *ad libitum* KDs showed an average 29 and 20% reduction in circulating IGF-1 and insulin levels, respectively (25). In summary, KDs have a broad array of promising applications from neuroprotection to cancer.

A potential obstacle to adopting a KD is however the experience of adverse symptoms in the first few weeks, often referred to as “keto-flu.” Symptoms of keto-flu may include headache, lightheadedness, fatigue, lethargy, “brain fog,” decreased exercise capacity, mood changes, constipation, muscle cramps, diarrhea and halitosis. Documenting such symptoms during keto-induction, and understanding the mechanisms behind them, might help prevent or relieve these temporary side-effects, as well as provide interesting physiological insight. We here therefore performed a scoping review of literature on the phenomenon of keto-flu, both with regards to an objective description of the condition, frequency of symptoms, the underlying physiology, and evidence for strategies of treatment and symptom alleviation.

## 2 Methods

### 2.1 Data selection

Embase, Medline and Web of Science electronic databases were searched online using a systematic search according to PRISMA guidelines for a scoping review, performed by two independent reviewers (Supplementary material). String one included various commonly used words and phrases relevant to the initiation phase of the KD: keto-flu, keto-induction, keto-adaptation and keto-adapted. In string two we linked these ketosis-related terms with several words referring to initiation or adverse effects. In string three we searched with the same initiation and adverse effects terms in conjunction with alternative words and phrases used to describe a KD or methods of initiating ketosis, for example: low-carbohydrate, fasting, starvation, carbohydrate restriction or low-calorie. To reduce the number of articles captured by the search, there had to be <2 words between the search words in the two parenthesis in string two and three (“adj2”). The same search used in Embase and Medline was adapted for use in Web of Science. In all the databases our search was constrained to the presence of relevant terms in the title, abstract and keywords.

In Medline and Embase the following search strings were used:

*1: (“keto-flu” or “keto-induction” or “keto-adaptation” or “keto-adapted”)*

2: ((Adaption or adaptation or induction or initiation or induced or initiating or inducing or onset or “adverse effect^*^” or complication^*^) adj2 ketosis)

3: ((Adaption or adaptation or induction or initiation or “adverse effect^*^” or complication^*^) adj2 (Ketogenic or “low-carbohydrate^*^” or “very-low-carbohydrate^*^” or VLCKD or “low-carb” or “high-fat” or fasting or “carbohydrate-withdraw^*^” or “carbohydrate-restricted” or “carbohydrate-free” or starvation or low-calorie^*^))

In Web of Science the following search strings were used:

*1: (“keto-flu” or “keto-induction” or “keto-adaptation” or “keto-adapted”)*

2: ((Adaption or adaptation or induction or induced or initiation or initiating or inducing or onset or “adverse effect^*^” or complication^*^) NEAR/1 ketosis)

3: ((Adaption or adaptation or induction or initiation or “adverse effect^*^” or complication^*^) NEAR/1 (Ketogenic or “low-carbohydrate^*^” or “very-low-carbohydrate^*^” or VLCKD or “low-carb” or “high-fat” or fasting or “carbohydrate-withdraw^*^” or “carbohydrate-restricted” or “carbohydrate-free” or starvation or low-calorie^*^))

### 2.2 Data eligibility

Two independent reviewers carried out a blind screening of the search results obtained using the inclusion and exclusion criteria detailed below. Articles were organized in Endnote and Rayyan. The reviewers discussed articles where they had conflicted views regarding inclusion. The reference lists of the included studies were also screened to include relevant additional articles missed by the initial search. On account of the heterogeneity of study design, observed population, objectives, outcomes and measures, a narrative review was deemed most suitable.

#### 2.2.1 Identified articles were included based on the following criteria

##### 2.2.1.1 Inclusion

1. Studies in which one of the study groups followed a ketogenic diet.
2. Studies observing short- term adverse effects of the transition to a ketogenic diet.
3. Studies examining possible mechanisms behind symptoms of keto-induction.
4. Studies examining possible interventions for relief of symptoms of keto-induction.

##### 2.2.1.2 Exclusions

1. Animal studies.
2. Studies not available in English.
3. Studies examining high-fat diets that are not low carbohydrate.
4. Studies exclusively studying the long-term benefits and side-effects of a ketogenic diet.

## 3 Results

**Figure 2:**
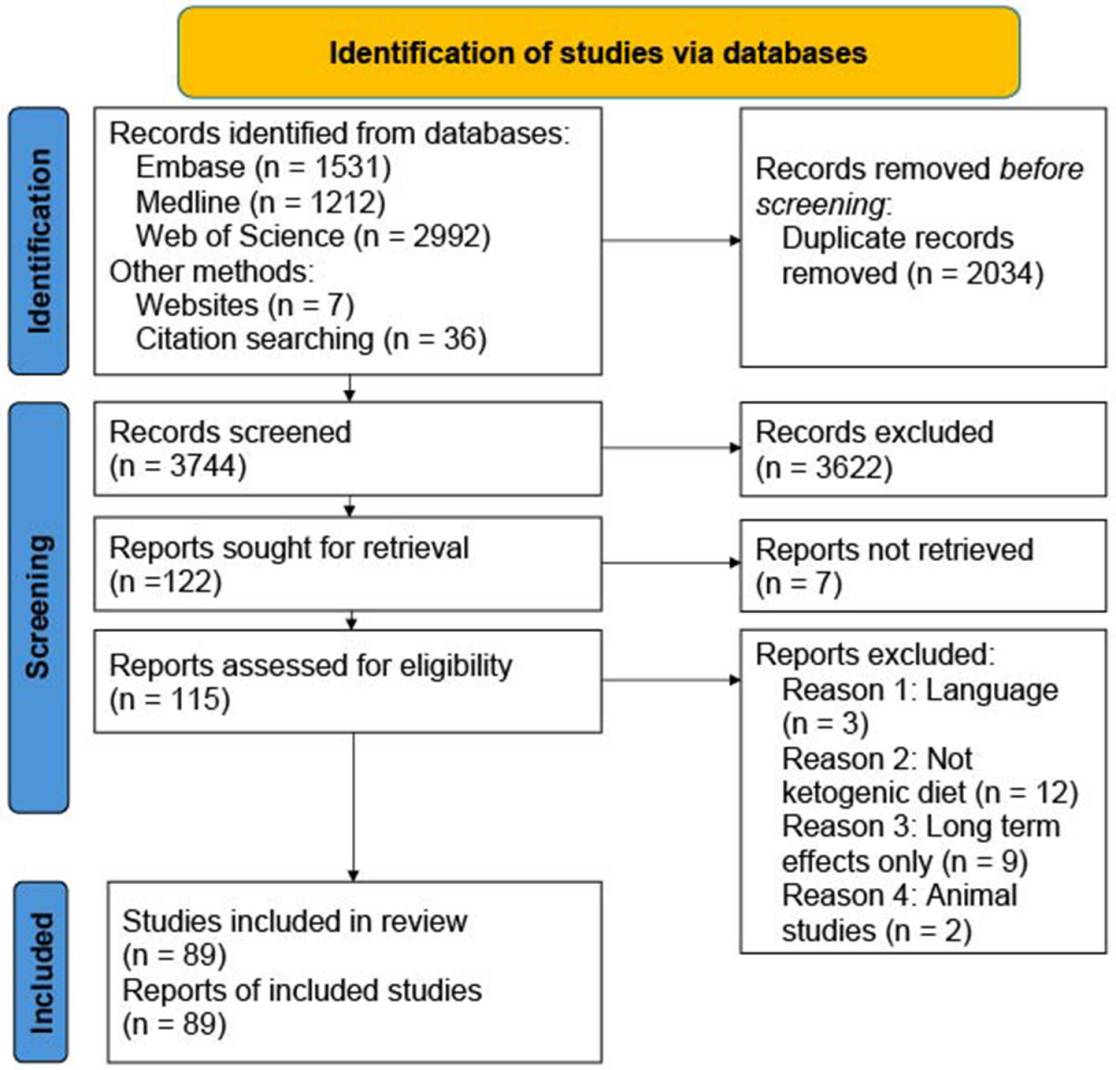
Overview of identified, excluded and included articles in the search.

The first search was performed on February 7, 2024 and a complementary search for new publications was performed on February 14, 2025. For the search in Embase, string one found 41 articles, string two 363 and string three 1,138, giving a total of 1,531 (Figure 2). In Medline, string one found 42 articles, string two 296 and string three 883, giving a total of 1,212 articles. Web of Science does not list the results per string, only total articles for the search which was 2,992. Among these 5,735 total hits we removed 2,034 duplicates, leaving 3,744 of which 3,622 were excluded based on title and abstract. Among the remaining 122 articles, 7 were not available in full text, yielding 115. In addition, three were excluded for not being available in English, 12 were excluded because the diets used were not ketogenic, nine were excluded because they only looked at long-term effects of the KD, and two studies were excluded because they were animal studies. This left 45 articles from our systematic search to be included in the study. We additionally included 36 articles from the reference lists of the articles from our systematic search. An additional seven articles were identified through web search which were used for comparative purpose in the introduction. This provided a total of 89 studies to be included in the study.

### 3.1 Symptoms of keto-induction

The identified articles describe that the transition from a standard diet high in carbohydrates to nutritional ketosis can involve a variety of transient symptoms, as summarized with reported occurrence rates in Table 1. These symptoms typically occur within 2–3 days of initiating a KD and most, if not all, resolve within 2–4 weeks, require no or minimal intervention, and are rarely so severe that they warrant discontinuation of the diet (16, 17, 24, 26). Reported symptoms of “keto-flu” included headache, light headedness, fatigue, lethargy, “brain fog,” decreased exercise capacity, mood changes, constipation, muscle cramps, diarrhea and halitosis (4, 5, 8, 27, 28). Other articles report additional symptoms including nausea, emesis, hypoglycaemia, acidosis, kidney stones and skin rash (26, 29, 30). Most of the data comes from the pediatric population, but similar symptoms are reported for adults and healthy populations as shown in the studies reviewed below. Generally, the studies included in this narrative review typically involve few participants with 5–100 participants and 10–30 in the average study, self-reporting, no control group, and heterogeneity in the type of KD between studies. All this heterogeneity makes it difficult to make precise estimates about the rate of occurrence for different populations and symptoms (17).

**Table 1:** Summary of reported prevalences of symptoms associated with keto-induction in the adult and pediatric populations.

**Symptom**	**Reported prevalence (adult population)**	**Reported prevalence (pediatric population)**
Halitosis	38%	
Constipation	1–68%	15–63%
Nausea	8–16%	27–42%
Vomiting	1%	5–36%
Diarrhea	2–23%	3.6–20%
Feelings of decreased energy	18–25%	17–28%
Liver function abnormalities	24%	
Light headedness/dizziness	15–21%	
Headache	8–25%	
Brain fog/reduced cognitive performance	10%	
Mood changes	1%	6.7–10%
Muscle cramps	3–37%	
Hypoglycaemia		6–28%
Keto acidosis		2–4%
Kidney stones	-	2.5–4%
Skin rash	13–21%	

Halitosis (bad breath) is described in some studies as a common side-effect of keto-induction, but not all studies report it as a side-effect, and only two have quantified its occurrence (5, 7). In the study that reported rate of occurrence, people on the KD experienced halitosis 38% of the time, compared to 8% on a low-fat diet (31). It has been reported at least in one study that the degree of halitosis is greater on a KD with lower carbohydrate intake and higher blood ketones (5).

Gastrointestinal adverse effects are amongst the most frequent symptoms of keto-induction. In the pediatric population constipation is reported to occur 15–63% of the time (14, 29, 30, 32). For the adult and healthy population constipation is reported in many studies as a “common side-effect,” but few studies have quantified the occurrence (17). Two studies respectively reported 27 and 1% occurrence of constipation in adults (4, 33). One study of adults comparing a KD to a low-fat diet for weight loss showed obstipation in 68 and 35% of participants on the respective diets (31). Nausea (27–42%) and vomiting (5–36%) are also common side-effects within the pediatric population (29, 32). For adults, nausea is reported to occur in 8–16% of participants and vomiting in 1% (4, 33). Diarrhea in the pediatric population is reported in three studies to occur 3.6–20% of the time, but three other studies do not report it as a symptom (14–16, 29, 30, 32, 34). Diarrhea among adults is reported in one study with 2% occurrence, or as a possible complication to intake of MCT oil (4, 17). In a study comparing a KD to a low-fat diet for weight loss, diarrhea occurred in 23% and 7% participants on the respective diets (31).

Another frequent symptom of keto-induction is feelings of decreased energy, usually described as fatigue, weakness, asthenia or lethargy. In the pediatric population it is reported to occur 17–28% of the time (29, 32, 34), while in adults 18% of the time (4, 33). In a study comparing a ketogenic and low-fat diet for weight loss, weakness occurred 25 and 8% on the respective diets (31). Moreover, light-headedness and dizziness are reported as relatively common transient symptoms of keto-induction in adults (15–21%), but are not reported for the pediatric studies. Also, headache is relatively frequent in adults (8–25%) but not reported in the pediatric literature. However, the pediatric literature reports fussiness, where one study estimated the prevalence to be 6% (4, 5, 14–16, 26, 29, 32–34). “Brain fog” or reduced cognitive performance has been reported to occur in 10% of healthy adults (4), and has been described as a “common symptom” by others (7). However, other studies have shown non-statistically significant changes in the ability to concentrate (5), and no difference between low-fat diets and KDs in terms of cognitive performance during a 6-week study with continuous testing (27). There have not been any reports of brain fog or reduced cognitive performance in the pediatric population (14–16, 26, 29, 30, 32, 34).

Reduced exercise performance and capacity while following a KD has been reported within the athletic community in several studies. It seems to be an expected side-effect for everyone on a KD, especially for anaerobic high-intensity exercise. The impairment seems to last 3–4 weeks, with return to normal exercise capacity and performance upon “keto-adaptation” for most if not all adults (35–38), also for high-intensity intermittent sprints (39). The adaptation period necessary has been coined “keto-adaptation” (30–33).

Mood changes are reported in several studies, and some studies have investigated effects of KDs on mood for different populations. In the pediatric population there is less data than for adults. For pediatric patients one study reports an occurrence of irritability of 6.7 and 10% for the KD initiated with a gradual increase in calories or at goal calories, respectively (34). For adults the results of the studies are somewhat heterogenous. Only one study (to our knowledge) has quantified the occurrence of “depressed mood,” reported in 1% of study participants (4). Multiple studies have shown a consistent improvement in mood from the start to finish of the diet (5, 7, 40). One study showed continuous improvement in mood, and no difference in mood scores if the diet was ketogenic, low-carbohydrate or moderately low-carbohydrate (5). Other studies have shown that reported “vigor-activity scores” (scoring of perceived energy) are higher on a non-ketogenic low-carbohydrate diet than on a ketogenic low-carb diet, meaning that people on the KD were less energetic during keto-initiation (41). One placebo-controlled study in healthy adults showed a transient increase in depression score at week 2 on a KD compared to a low-fat diet or a KD with ketone salts (27). Week 2 after starting a KD would be in the middle of the “keto-flu”/keto-induction period.

Muscle cramps are a common symptom of keto-induction for the adult population, but there are no reports of it in the pediatric literature that we have identified (14, 16, 26, 29, 32, 34). For the adult population muscle cramps is reported to occur in 3–37% of study participants (4, 31, 33). In a study comparing a KD to a low-fat diet the occurrence of muscle cramps was 37 and 7% on the respective diets (31).

Hypoglycaemia is a potential side-effect that appears to mainly affect the pediatric population. There have been no reports of hypoglycaemia within the adult population in the studies we have identified, even though it is widely accepted that KDs produce significant reductions in blood glucose within the normal range. Typically, the adult population is less monitored during the induction phase compared to pediatric patients. Therefore, there are less available measurements for the keto-induction period in adults. Some studies report symptoms like shaking (5%), irritability (3%), fatigue (18%) and dizziness (15%) which could be symptoms of hypoglycaemia in adults, but it is difficult to evaluate in the absence of systematic glucose measurements (4, 5, 7, 8, 33, 40, 41). Three studies in adults report a marked drop in blood glucose for KDs compared to a low fat, medium-carbohydrate diet or standard diet, but report no symptoms of hypoglycaemia (27, 42, 43). For the pediatric population, hypoglycaemia is a known side-effect of keto-induction which also sometimes requires simple treatment. The occurrence is reported to be 6–28%, and some studies report recurring episodes of hypoglycaemia to be present 17% of the time (14–16, 29, 30, 32, 34).

Acidosis is also a potential side-effect primarily observed in pediatric patients. There are no reports of adults without diabetes type 1 or other types of endocrine pathology that report acidosis in the keto-induction period (4, 5, 7, 8, 33, 40, 41, 44). In the pediatric population the occurrence of acidosis is reported to be 2–4% in the keto-induction period (14, 29, 32, 34).

Kidney stones are a frequently cited side-effect of the KD. This is not strictly a side-effect of keto-induction, since kidney stones usually occur more than 4 weeks after initiation of the KD. However, the physiological alterations that predispose individuals to kidney stones may happen during the keto-induction phase. These physiological alterations are increased acid load to the kidneys, hypercalciuria and high phosphate in urine (8, 15, 44). In the pediatric population the occurrence of kidney stones has been reported as 2.5–4% with up to 25% in long-term adherence to the KD (15, 26, 29, 45). None of the studies looking at short-term side-effects report kidney stones. Studies in adults do not report kidney stones, but the same physiological adaptations that could predispose to kidney stones are seen (4, 5, 8, 33, 41, 44).

Another of the cited symptoms of “keto-flu” is skin rash, but only reported in two of the identified studies (7, 18). One study compared a KD to a low-fat diet and found that skin rash occurred in 13 and 0% of study participants on the respective diets (31). Another study found a skin rash frequency of 21% during keto-induction (7). There is unfortunately no further expansion on the details of the skin rash involved. However, prurigo pigmentosa is a skin condition that has been associated with a ketogenic state following either induction of a KD, dieting, anorexia, diabetic ketoacidosis or bariatric surgery. In the popular media prurigo pigmentosa has been coined “keto rash” (46–49). However, this is a rare condition, as described in numerous case studies (50, 51). In the pediatric literature on KDs there are no reports of skin rash (50).

### 3.2 Proposed mechanisms behind keto-induction symptoms

#### 3.2.1 Natriuresis, kaliuresis and hypovolemia

The physiological adaptations that occur during a KD resemble those of fasting or starvation ketosis. The low availability of dietary carbohydrate leads to a reduced blood glucose level, mimicking a starvation state. In a starvation state, energy is derived from fat and protein from endogenous sources like adipose tissue and muscle. A KD mimics this state by providing minimal dietary carbohydrate but adequate protein and fat to avoid a catabolic state (43, 52). By maintaining a normal energy intake, KDs will also produce some different responses than fasting/starvation. Generally, however, low intake of dietary glucose decreases 24-h insulin secretion by over 50%, while there is a compensatory increase in fat oxidation to avoid hypoglycaemia (43).

Insulin has well-documented anti-natriuretic effect on the human kidney, which increases sodium reabsorption in the presence of elevated insulin (53–55). In the 1960s and 70s the first studies on human starvation and fasting showed that the fasting state produced a profound natriuresis, kaliuresis, diuresis and consequential loss of body water (52, 56, 57). Upon both fasting and KD initiation there is an initial rapid loss of body weight which is predominantly from loss of water secondary to natriuresis (8, 52, 57–59). The secretion of sodium and potassium is greatest between days 1–4, and has been shown to promptly stop after carbohydrate administration (55, 56). The loss of sodium has been coined “the natriuresis of fasting” (58). The results have been replicated during KD initiation as in fasting (52, 57). Accordingly, studies have shown that fasting and keto-induction significantly reduce serum sodium and potassium levels (44, 56, 58), and that the increased natriuresis and kaliuresis subside after 14 days on a KD, which is also observed during fasting (56, 57).

The symptoms from continued sodium loss and the following minor hypovolemia can be dizziness, orthostatic hypotension, fainting, fatigue, constipation, and headache (60), many of which we can recognize are frequently observed symptoms of “keto-flu.” Hyponatremia also has a known association with depressed mood (61, 62). Furthermore, kaliuresis could be a secondary consequence to natriuresis triggering aldosterone secretion which stimulates the kidneys to conserve sodium at the expense of potassium. Negative potassium balance can present clinically as muscle twitches, constipation, cramps, irregular heartbeats or muscle weakness [neuromuscular dysfunction; (60)], many of which have been observed during keto-induction. It should be noted that we have not identified any single study that documents both symptoms of keto-induction and concomitant changes in serum/urinary potassium, sodium, or loss of body water, so a causal relationship needs to be confirmed.

#### 3.2.2 Reduced energy substrate availability

Metabolic adaptation toward increased lipolysis and ketosis on a KD does not occur instantaneously, and hence there is a window where dietary glucose is restricted while ketone bodies are not yet readily available. Harber et al. carried out a study on glucose kinetics using gas exchange and stable isotope analysis of blood plasma prior to and during the first 7 days following commencement of a eucaloric carbohydrate restricted [under 5 energy percent (E%)] KD (43). They observed an acute 20% reduction in glucose during the first 2 days of carbohydrate restriction, before a return to pre-dietary levels on day three. But more interestingly the analysis of glucose kinetics showed that endogenous glucose production and systemic glucose uptake decreased suddenly after day 1 of carbohydrate restriction, whereas carbohydrate oxidation decreased more gradually. However, after 1 week the whole-body glucose oxidation was suppressed to a greater degree than the whole-body glucose uptake, meaning that an increased proportion of the total glucose taken up in cells goes to glycogen synthesis rather than glucose oxidation. It was observed that muscular glycogen levels decreased 20% after 1 week. As individuals in the observed test population were sedentary, Harber et al. postulate that the observed increased glycogen synthesis must be occurring primarily in the liver, providing a potential means of maintaining euglycemia in extended fasting (43). These results were an extension to previous research using simpler techniques, however there were some notable incongruities with previous observations. Nilsson et al. reported more than 50 years ago that liver glycogen was almost completely depleted after 24 h on a eucaloric low-carbohydrate diet, in a trial using percutaneous liver biopsies (63). It is elsewhere shown that although the rate of hepatic glycogenolysis during carbohydrate restriction is high enough to completely deplete hepatic glycogen stores in 1 day, hepatic glycogenolysis still provides roughly a third of total glucose production after 3 days of fasting, and thus it is unlikely that liver glycogen stores are completely depleted under short-term carbohydrate restriction (43).

Notably, the studies we have reviewed showing hypoglycaemia upon keto-induction are primarily on children. It is proposed that this is due to children having smaller glycogen reserves that are more rapidly depleted under dietary restriction (64). A study by Bergqvist et al. showed that children were significantly more likely to experience hypoglycaemia in a monitored 6-day KD introduction, when the diet was initiated with a < 48 h fast compared to a gradual increase in the dietary fat: carbohydrate ratio [33% compared to 4% for the respective dietary approaches; (64)]. Nonetheless, the extent to which hypoglycaemia or mildly reduced blood glucose levels play a role in some of the more subjective “keto-flu” complaints is unclear. Common symptoms of mild hypoglycaemia, such as fatigue, concentration difficulties, paleness, shaking, paraesthesia, headache, hunger/thirst, changes in behavior, restlessness, dizziness and muscle weakness, are at least partly shared with reported symptoms during keto-induction. However, the pediatric studies of keto-induction do not report the symptoms of hypoglycaemia, only occurrence of objectively measured hypoglycaemia. The lack of more direct evidence linking transient hypoglycaemia to symptoms of keto-flu calls for more studies in both children and adults.

The reported reduction in exercise performance and capacity during the first week of keto-induction may also involve reduced energy substrate availability, as glucose oxidation, muscle- and liver glycogen and systemic carbohydrate availability are reduced (36, 38, 43, 65). Studies have shown normalization of performance/capacity after a 4-week adaptation period, at least for aerobic exercise intensities (21, 35, 36, 38, 66, 67). During this adaptation period, termed “keto-adaption,” maximal fat oxidation has been shown to increase 2-to-3fold, ketolysis and lipolysis increase, and after 20 months of adaptation there was no difference in muscle glycogen stores. In the same adaptation period it has been shown that glucose oxidation is also reduced 2-to-3fold (21, 37, 65, 66). Whether a reduced exercise performance and capacity during keto-induction involves other mechanisms than reduced energy substrate availability remains uncertain, although it appears plausible that slight hypovolemia in myocytes due to water loss in natriuresis might also contribute. Of note, a recent study found that a low-carbohydrate high-fat (LCHF) ketogenic diet did not reduce time to exhaustion during prolonged endurance exercise compared to a high-carbohydrate diet, and that addition of carbohydrates after 4-week keto-adaptation to the LCHF diet improved performance compared to placebo (67).

#### 3.2.3 Delayed gastric emptying and fat malabsorption

An effect of KDs, with their high fat content, is to slow down gastric emptying time by increasing cholecystokinin secretion which reduces gastric motor function (68). When changed more suddenly, this can trigger nausea and vomiting (69). Transient diarrhea is in the pediatric literature proposed to result from defective absorption of or intolerance to fat, and thus should disappear when the GI system is given enough time to adapt to the high fat content of the diet (15, 69).

#### 3.2.4 Altered liver function

In an analysis of 25 patients in routine practice who were treated with a KD, 24% showed acute, asymptomatic changes in liver function tests during the initial days/weeks, in both hepatocellular (disproportionate elevation in aspartate aminotransferase and alanine aminotransferase) and cholestatic patterns (disproportionate elevation in alkaline phosphatase, γ-glutamyl-transferase, and bilirubin), with or without the concurrent use of hepatotoxic medications (70). In most cases, the ketogenic diet could be successfully continued by adjusting medications, and in some instances the KD also lead to acute improvements in chronically elevated liver function tests. In the case of a cholestatic pattern, patients received choline supplementation while continuing the KD. Further research is needed to determine if the abnormalities can resolve on their own while on the ketogenic diet, or if routine choline supplementation should be considered. Taken together, the authors conclude that monitoring liver function tests is crucial during the acute phase of ketogenic diet initiation (70).

#### 3.2.5 Hyperketosis

Nutritional ketosis is typically defined as dietary-induced circulating βHB level >0.5 mmol/L, however the presence of considerably higher levels, up to around 2 mmol/L, have been observed by Harvey et al. under induction of the KD in conjunction with MCT supplementation (7). In another study monitoring the effects of differing levels of carbohydrate restriction, it was shown that participants on a diet constituting of 5 E% carbohydrate had close to double the level of βHB than those on a diet with 15 E% carbohydrate (5).

The higher levels of βHB observed by Harvey et al. in their carbohydrate level study was also statistically significantly associated with an increased level of halitosis, but they found no association to mood (5). In the study using induction with MCT, however, there was a strong inverse correlation between βHB and mood disturbance (7). In extremely anomalous cases or where there is underlying pathology it may be possible to see levels of ketosis that are approaching those seen in diabetic ketoacidosis (71). Mir et al. mention in the description of their protocol for initiation of KD in children with epilepsy that they observe patients for signs of hyperketosis such as rapid panting, increases heart rate, facial flushing, irritability, vomiting and unexpected lethargy (16).

#### 3.2.6 Changes in urine pH, calcium and phosphate

As stated previously, kidney stones do not typically arise under keto-induction, but metabolic alterations that predispose individuals to kidney stones do occur. Kidney stones are primarily seen in the pediatric population, but the same initial metabolic alterations are also seen in adults. Kidney stones during a KD have been reported to consist of uric acid, calcium oxalate or an amalgamation, together with phosphate (15).

During keto-induction there is an increased acid load to the kidneys, hypercalciuria and high urine phosphate. The increased acid load probably comes from protonation of ketones [ketones are produced as conjugate bases, not acids; (72)], and the increased intake of sulfur-containing amino acids as KDs is often (but does not have to be) higher in animal proteins. Oxidation of sulfur to sulfate generates protons. Low urinary pH predisposes for uric acid precipitation since the pKa of uric acid is 5.7, therefore the solubility of uric acid decreases with low pH, and thus increasing the risk of kidney stones (8, 44). Some studies have also shown an increased excretion of uric acid in the keto-induction phase, which would amplify the risk even further (15).

Hypercalciuria has also been documented following keto-induction, with some studies documenting a 60% increase in excreted calcium, which could increase the risk of precipitation and developing kidney stones (44). Some studies have documented that this effect is transient, with hypercalciuria 2 weeks after keto-induction, but not after 6 weeks (8). Hypercalciuria is proposed to be secondary to increased acid excretion in the kidneys (73). Low intake of fluids will increase the urine concentration of calcium and uric acid even further, and thus increase the risk of developing kidney stones (74).

#### 3.2.7 Changes in microbiome

Indigestible carbohydrates (fiber, oligosaccharides and resistant starch) provide the basic energy source for microbes. With the major changes in dietary macronutrient composition that occur in the transition to a KD, there may be substantial changes in the environment for gut microbes. Studies of both rats and humans have shown that induction of the KD leads to a reduction in both abundance and diversity of gut microbes (10, 65). However, a study in patients with multiple sclerosis (MS) starting on a KD showed a significant increase in bacterial abundance at 6 months after an initial decrease at 2 weeks, suggesting this adaptation is dynamic and that the gut microbiome may play some role in keto-adaption (65).

A study into the microbiome of race walkers consuming a KD for 3 weeks compared to a high-carbohydrate and periodised carbohydrate group showed not only changes in the intestinal microbiome but also to their oral microbiome (65). In the KD group there was a relative decrease in the quantity of *Haemophilus, Neisseria*, and *Prevotella spp*. and an increase in *Streptococcus spp*. Interestingly it was speculated that these changes produce an environment that compromises the enterosalivary nitrate-nitrate-NO axis, which would theoretically affect exercise performance in a negative manner (65).

A KD is associated with a lower intake of fiber as one excludes starchy vegetables and fruit. The intake of non-starchy vegetables also needs to be moderate to allow sufficient carbohydrate restriction and thereby ketogenesis (60). The reduced intake of fiber has been tied to increased occurrence of constipation, which is a symptom of keto-induction (15, 26). However, there is opportunity for large variations in food selection, and consequently intake of indigestible carbohydrates, on different variants of a KD. Thus, it is difficult to compare how different KD formulations affect the microbiome based on macronutrient distribution and between studies. There appear to be no controlled studies on the impact of dietary fiber in the context of a KD (60). One can assume that eating a KD high in indigestible carbohydrates has a more beneficial effect on the microbiome, and that the amount of indigestible carbohydrate is more important for the microbiota than the state of ketosis by itself.

#### 3.2.8 Prurigo pigmentosa

Prurigo pigmentosa (PP) is a rare skin rash characterized by pruritic, erythematous papules and vesicles with a reticulated presentation on the back, chest and neck (46). Although most frequently diagnosed in Japan, there have been cases diagnosed worldwide in patients of other ethnicities, including some predisposed individuals identified in Turkish and Sicilian populations (49). The pathology of the condition remains uncertain but there appears to be an association to a state of ketosis. In several case reports, the onset of PP coincides with dieting, KD initiation, fasting, weight loss, anorexia nervosa and diabetes mellitus. Additionally several case reports show spontaneous resolution following reintroduction of dietary carbohydrates and ketone level normalization (49).

A proposed pathophysiological mechanism for the possible link between ketosis and PP is based on the passage of ketone bodies from the bloodstream into the perivascular space and induction of cytotoxicity and inflammation. Increased levels of ketone bodies have been shown to cause an upregulation of both intercellular adhesion molecule 1 (ICAM-1) and leukocyte function antigen 1 [LFA-1; (49)]. These same molecular changes have been observed in the lesional keratinocytes in PP. The interaction of increased ICAM-1/LFA-1 is believed to be the primary pathway by which leukocytes can attach to keratinocytes and endothelial cells, thus allowing for tissue migrations and specific immunological reactions. Individual keratinocyte strains show great variability in the expression of ICAM-1, likely due to genetic polymorphisms and hence perhaps explaining some of the observed ethnogeographic predisposition (49).

Much of the literature on prurigo pigmentosa and its possible link to ketosis is contained in a handful of small case report reviews, and the proposed pathophysiology appears plausible but somewhat speculative. However, the observation that reintroduction of carbohydrates leads to remission strengthens the hypothesis that PP is related to ketosis.

### 3.3 Predictors of adverse effects

Although the underlying physiological adaptions that take place following initiation of a KD initiation are universal, and most people report some level of symptoms, there are some populations that appear to be more susceptible to adverse symptoms. Lin et al. found age to be a statistically significant predictor of adverse effects in their study of KD complications in epileptic children, supporting that younger children appear to be particularly susceptible. Age was also significantly correlated with hypoglycaemia upon fasting initiation (32). Mir et al. also concluded that children under 3 years of age carried a greater burden of adverse effects than adults and children over 3 years old (16). Interestingly, however, Armeno et al. studied the effectiveness and tolerability of a KD in infants under 2 years of age with drug resistant epilepsy, and found that when initiating the diet without a fast the diet was both tolerable and effective for this group. They did however observe an increased rate of acute adverse events in infants younger than 1 year old (14). Based on the same study, Mir et al. observed that symptoms were predicted by not only age but also body weight, concluding that underweight children are more at risk of adverse outcomes (16).

The literature proposes that a KD is contraindicated for certain groups of patients, including those with anorexia and pancreatitis (75) as well as a range of metabolic disorders including primary carnitine deficiency, carnitine palmitoyl transferase deficiencies, carnitine translocase deficiency, beta-oxidation defects, pyruvate carboxylase deficiency and porphyria (16, 26). In a case study of KD-induced ketoacidosis in a non-diabetic lactating woman, Bedock et al. propose that caution should be practiced in the case of application of the KD to post-partum lactating women due to the increased energy demands of breastfeeding (76). A KD is also contraindicated in patients who will be receiving a propofol infusion due to risk of developing the potentially fatal propofol infusion syndrome (26).

### 3.4 Symptom mitigation and treatments

In an effort to ease the transition to a state of nutritional ketosis, a number of strategies have been studied and presented in the literature. Several metrics are used when considering the effectiveness of a given intervention, both related to the mitigation of adverse effects and in the time required to reach ketosis (18). Here we will present proposed strategies and their rationale, for the following three categories which broadly represent the available literature: Alternative KD formulations, differing methods of initiation, and dietary supplements.

#### 3.4.1 Ketogenic diet formulations

The classic KD is composed of a 4:1 ratio of fats to non-fat (carbohydrate and protein combined) calculated by molecular mass (17), however it is worth noting that in some studies diet compositions are referred to by E% from fat and/or carbohydrate of total energy. There also exist a multitude of 3–4 letter abbreviations that describe KDs that should not be directly compared between studies without closer examination of the applied definitions.

Adjustments of KD compositions have often been made to increase food choice flexibility, feasibility and palatability, such as less strict carbohydrate and protein restriction while still achieving nutritional ketosis. An example of such a diet is the modified Atkins diet, where the fat to carbohydrate and protein ratio is closer to 2:1 and the ratio of protein to carbohydrate is also 2:1 (26). The higher carbohydrate or “modified ketogenic diets” are often supported by supplementation with MCT which have been demonstrated to aid both ketonemia and ketogenesis (18).

Harvey et al. performed a randomized controlled trial to study the effect of differing levels of carbohydrate restriction on symptoms of carbohydrate withdrawal and mood, and whether less restrictive diets can elicit NK, defined as ≥0.5 mmol/L βHB, without the aid of supplementation. They observed that ketonemia was proportional to the level of carbohydrate restriction and that NK was consistently achieved in the groups receiving a diet of 5 E and 15 E% carbohydrate, but only sporadically in the group receiving 25 E% carbohydrate. Time to ketosis was however slightly quicker for the 5 E% group, being 4 days rather than 5 days for the others. An overall sum of symptoms score was recorded for all groups each day under diet initiation, and only the 5 E% carbohydrate group had a statistically significant change from baseline but the difference between groups was overall not significant. However, when considering individual symptoms, muscle weakness and halitosis were higher in the 5 E% carbohydrate group. These results imply that although serum βHB levels are increased proportionally with carbohydrate restriction, satisfactory NK can be achieved with a less restrictive diet while easing some initiation symptoms.

Volek and Phinney et al. make the case for a well-formulated KD whereby there is also close consideration of the foods consumed. The specification of a macronutrient ratio still leaves considerable freedom to choose the sources of fats, proteins and carbohydrates, and not least the micronutrient content of foods consumed. Management of insulin resistance may be improved by swapping high-glycaemic, highly processed, nutrient-depleted carbohydrate sources in favor of lower-glycaemic, nutrient-rich, whole foods. When it comes to fats, concentrated sources of polyunsaturated fats are not well tolerated at the quantities of fat consumed in a KD, with increased gastrointestinal complaints; saturated fats and monounsaturated fats seem to be better tolerated (60).

#### 3.4.2 Methods of initiation

How a drastic dietary change is implemented affects tolerability. Traditionally, initiation of the KD for use in pediatric epileptic populations has utilized an initial period of fasting of 24–48 h, followed by a gradual increase in caloric intake and/or fat: carbohydrate and protein ratio (34, 77). Fasting has historically been widely considered beneficial due to the rapid achievement of ketosis, giving quick and effective control of seizures. A fasted initiation has been shown to have a statistically significant shorter time to the achievement of ketosis than a gradual transition (26). However, several studies have shown that long-term efficacy in terms of seizure control is not improved by initiation with a fast relative to gradual initiation (26, 64). On the other hand, children experience a greater burden of adverse effects in the form of hypoglycaemia and lethargy under a fasted initiation regimen (26, 32). The burden of hypoglycaemia in a fasted initiation is significantly correlated with age, whereas lethargy is not (32). It is broadly agreed in the literature that a fasted initiation is not necessary as it has little clinically significant advantage for seizure control, and as it leads to an increased burden of unwanted side-effects and hence reduced tolerability (32, 64, 77).

When not initiated with fasting there are several alternative strategies of initiating a KD. These involve either initiation of the KD at full energy content with the intended permanent fat: carbohydrate and protein ratio, or alternatively a gradual increase of this ratio, either at predicted full energy content or at a gradually increasing caloric content over the first 4–6 days. Two examples of gradual initiation protocols are those used by Bergqvist et al. and Bansal et al., respectively. Bergqvist started their group on their full caloric requirement, increasing from 1:1, 2:1, 3:1 and 4:1 grams fat to carbohydrate and protein over the first 4 days (64). Bansal et al. started their group at 4:1 grams fat to carbohydrate, while the caloric content increased from 33%, 67% and then 100% over the first 3 days of the diet (34). There is little literature comparing these various forms for gradual initiation, but Bansal compared their gradual caloric advancement group with initiation at full calories on a 4:1 grams fat: carbohydrate diet. They found that the gradual initiation group reached nutritional ketosis earlier, but although this difference was statistically significant it is unlikely to be of clinical significance given that the mean time to ketosis was on the same day [day 2.93 vs. 2.17; (34)]. Tolerability was similar for both groups with no statistically significant differences in reported adverse symptoms, although a greater proportion of the 100%-calorie group was started on sodium citrate during their hospitalization (34). Average carbon dioxide between groups however did not differ significantly, suggesting that the use of sodium citrate is not necessarily indicative of increased acidosis. Interestingly, the 100%-calorie initiation group appeared to exhibit better seizure control at both 1- and 3- months post-initiation, although it should be noted that the groups were small and several patients left the trial before this point in the study. It was concluded that starting the KD at full caloric load appears as a safe and effective option (34).

#### 3.4.3 Dietary supplements

The strategies proposed in the literature with respect to dietary supplementation vary, with some being aimed at particular symptoms and others being more targeted to the underlying process of ketosis or observed physiological adaptations that occur during keto-induction.

##### 3.4.3.1 Electrolytes

Consumption of electrolytes is heavily encouraged to compensate for the natriuretic and kaliuretic effects of a reduced insulin level (27, 60, 78). In the identified literature there is however little rigorous study and quantification of this requirement. Volek and Phinney advise drinking 1–2 cups of broth or bouillon per day whilst on a low-carbohydrate diet, with an additional serving 1 h prior to exercise. They note that this provides 1–2 grams of extra sodium to the diet and should be enough to mitigate symptoms of weakness, fatigue and hypotension associated with hypovolemia and natriuresis. They also recommend an intake of 4 g/day of potassium, mainly through bone broth or vegetables like avocados, nuts and seeds (78). Kackley et al. carried out a study on the effect of KD on mood states, both with and without the addition of exogeneous ketone salts. Ketone salts provide, in addition to βHB, sodium, calcium and magnesium. A marked improvement in depressions scores was observed in the group consuming ketone salts in addition to a KD, but there remains ambiguity over the causation of the observed improvement as ketone salts supply both ketones and electrolytes. It was proposed that further research should be carried out with sodium-matched groups (27).

Hyponatremia, hypokalaemia and secondary hypovolemia can also predispose individuals for constipation. No studies were identified that test if supplementing with sodium, potassium or a liberal fluid intake reduces occurrence of constipation in relation to keto-induction. However, some authors recommend supplementing with sodium and potassium to avoid constipation and other symptoms of keto-induction (60), and liberal fluid intake is a common medical recommendation for avoiding/treating constipation as it can soften the feces.

##### 3.4.3.2 Carbohydrates and dietary fiber

Whilst the supplementation with carbohydrates during a KD could be likened to supplementing a vegetarian diet with bacon, fine-tuning of the carbohydrate content of the diet can help adjust levels of ketosis (5). Mir et al. used urinary ketone levels to guide the progression of their gradual introduction to the KD (16). The diet was started at 2:1 fat to protein and carbohydrate and progressed or regressed based on individualized follow-up with consideration of satisfactory urinary ketone levels and monitoring for signs of acidosis, excess ketosis and hypoglycaemia. Lin et al. monitored symptoms and used juice consumption for treating hypoglycaemia in children during KD initiation, and found that the juice helped prevent hypoglycaemia and lethargy in 24% of their studied cases, while a change in the KD ratio was used only in 2% of cases (32). This suggests that targeted addition of carbohydrates during KD induction can be a practical way to adjust the level of ketosis and thereby avoid excess symptoms.

Constipation is a common symptom of keto-induction that occurs in 15–63% of the pediatric population, and 1–27% among adults. Aside from adequate supplementation of sodium and potassium with liberal fluid intake, increasing fiber intake is a proposed treatment/prophylaxis to avoid constipation in many studies (4, 15, 17, 75). Increasing fiber intake can be done through deliberate food choices such as increasing the intake of non-starchy vegetables, nuts and seeds, fiber-based products like psyllium husk, or medical fiber-containing laxatives (15, 60). However, no studies have experimentally documented that increased fiber reduces the occurrence of constipation in relation to keto-induction. Nonetheless, increased fiber intake is a common general medical practice for constipation. Vollek and Phinney note that a fiber intake of 15–20 g per day in the form of non starchy vegetables, nuts and seeds appears to be sufficient (60).

##### 3.4.3.3 Medium-chain triglycerides

Medium-chain triglycerides (MCT) are commercially available inexpensive dietary supplements with proven potential to induce ketosis, with or without the aid of the classic KD (79). MCT have become a popular addition to KDs since it can allow for greater intake of carbohydrates and protein, while maintaining similar levels of ketones and efficacy in treating epilepsy (29). Studies have shown that MCT supplementation can increase palatability, tolerance, and compliance (26, 45). MCT can be used intermittently to spike ketones, or as a fixed component of the KD (79). Studies have shown that MCT can increase ketones in a near-linear dose-dependent manner when the right form of MCT are administered [C8 and C10; (79)]. A unique property of MCT is that they can be absorbed directly through diffusion into the hepatic portal vein, and do not rely on bile or micellar-chylomicron mediated absorption through the lymphatics like long-chain triglycerides. After absorption, MCT are preferentially converted into ketones in the liver (7, 18). MCT are triglycerides where two or three of the fatty acid chains attached to the glycerol backbone are medium in length, which means 6–12 carbons. Based on the carbon-chain length (Cx) the fatty acids have different names and metabolic effects. The different MCT types are: Caproic- (C6), caprylic- (C8), capric- (C10) and lauric acid (C12). The ketogenic effect of C8 is three times higher than C10, and six times higher than C12 (18). Therefore, some studies recommend consuming only C8 or a combination of C8 and C10 (79).

MCT supplementation has been proposed as a possible intervention to alleviate symptoms of keto-induction. The rationale for MCT supplementation is that during keto-initiation serum glucose concentration is reduced for up to 1 week, while there is also limited production of ketones. Therefore, there is a lack of substrate for energy which could cause symptoms of keto-induction as before mentioned. Thus, MCT supplementation has been proposed as a potential energy source to fill the gap of missing energy substrate, while also allowing for continued adaptation to burning fat as the primary fuel (7). Ingestion of 10–15 g of a C8 and C10 mixture increases plasma ketones to 0.5–1.0 mmol/L for about 5 h. Serum ketones are elevated 30–60 min after ingestion. If one ingests 10–15 grams three times a day one would remain in mild ketosis throughout the day (18, 80). MCTs are about as potent as ketone salts in raising βHB (22). When comparing the keto-induction of a 4:1 KD with and without MCT, the MCT group had 0.2–0.7 mmol/L higher βHB levels with 30 g C8 (65%) and C10 (35%) compared to the non-MCT group (7). There was however no significant difference in time to ketosis between the diets. Studies have used from 5 to 60 g of MCT, and MCTs are reported to be safe up to 1 g/kg (18, 79, 80). Furthermore, to increase the ketone production from MCT they should be consumed without a meal, if tolerated, or together with a low-carbohydrate meal. Consuming glucose and MCT together was found to reduce the ketogenic effect of MCT, whilst consumption of caffeine together with MCT could potentiate the ketogenic effect (79).

Harvey et al. (7) studied symptoms by a “symptom score” of the most frequently reported symptoms. Symptoms initially worsened in response to both diets. In the following days symptoms were reduced in the MCT group relative to the non-MCT group with a “possible beneficial clinical effect” across all time points. The MCT group saw improvements in concentration difficulties, muscle cramps, intestinal bloating and constipation. The non-MCT group reported significant factor increases of 0.5 for concentration difficulties, 2.0 for muscle cramps, 1.7 for intestinal bloating and 2.3 for constipation. Abdominal pain was significantly higher by a factor of 1.7 in the MCT group, as one would expect from the known side-effects of MCT. Both groups saw improvements in mood (7).

While laxative effects of MCT supplementation may offset constipation, there may also be side-effects, in particular gastrointestinal distress like discomfort, nausea, diarrhea and vomiting when consumed in large amounts (79). Such side-effects were found to be dose-dependent, but it is possible to develop tolerability to higher doses with gradual titration (79). For example, at doses of 50–60 g, 100% of participants were reported to experience gastrointestinal distress, whereas with 30 g only a few participants experienced mild gastrointestinal effects (81). To avoid side-effects it is recommended to start with 5 g of C8 or C8 and C10, and then titrate the dose up when gastrointestinal issues no longer occur. The goal is 15–20 g of C8, where one is to expect mild ketosis and minimal side-effects. Side-effects can also be reduced by emulsifying the MCT oil (81).

Taken together, although the available data and physiological rationale suggest benefits of titrated MCT supplementation, with increased ketone production to provide energy substrate when glucose availability drops, more controlled studies are needed to clarify the pros and cons of MCT for symptom reduction during keto-induction.

##### 3.4.3.4 Leucine/lysine

Leucine and lysine are amino acids that do not contribute to gluconeogenesis, and are thus considered to be solely ketogenic (18). The progression of fasting leads to an increased conversion of leucine into ketone bodies as peripheral tissue is catabolized to feed leucine into this process. It has therefore been proposed that supplementation with leucine may bolster ketogenesis. However, there is no clear evidence that leucine supplementation significantly increases circulating βHB levels (18). Our review has found no investigation into the use of lysine supplementation for this purpose.

##### 3.4.3.5 Exogenous ketones

Exogenous ketones are a means of providing βHB directly to the body with neither the requirement for ketogenesis nor elevation in circulating free fatty acids (18). By supplementation, βHB can reach the same circulating concentrations as seen under endogenous nutritional ketosis but within minutes, regardless of carbohydrate restriction (66). Supplements can be in the form of ketone salts, where βHB is bound to a combination of magnesium, sodium, calcium or potassium, and are typically sold as a racemic mixture of D and L enantiomers, where a typical dosage will raise the blood concentration of the metabolically utilized D-βHB to 0.3–1 mmol/L. The non-physiologic L enantiomer can be activated in the liver mitochondria and converted to physiological ketones (22). These ketone salts do however also increase the salt load, which can reduce tolerability in the form of gastrointestinal distress, acidosis and cation overload (18, 66). However, in instances where ketone salts are taken as a supplement to a KD, this salt load may compensate for the increased natriuresis and kaliuresis experienced under the initiation of the KD, thus potentially alleviating symptoms during keto-induction (27).

Alternatively, exogenous ketones can be consumed in the form of ketone esters, which are either in the form of R,S-BD AcAc diester or R-BD D-βHB monoester, where both are hydrolysed by carboxylesterases and esterases situated primarily in the gastrointestinal tract. The metabolism of R,S-BD AcAc diester produces a comparable rise in D-βHB to that observed with ketone salts, but this ketone ester is accompanied by a significant rise in blood AcAc (~0.4 mmol/L). R-BD D-βHB monoester metabolism on the other hand has been shown to increase the blood D-βHB concentration to 2–5 mmol/L (66). An examination of the pharmacokinetics of ketone salt vs. ketone ester supplementation has shown that although they reach similarly significant concentrations of D- βHB, T_max_ for ketone salts is around 90 min whilst only 50 min for ketone esters. In other words, it takes almost twice as long to reach a maximal D-βHB concentration in the bloodstream after ingestion of ketone salts. In both cases baseline concentrations of D-βHB was reached again after 3–4 h post-ingestion (22).

In 2020 Pimentel-Suarez et al. carried out a safety and tolerability evaluation of a third means of inducing ketosis exogenously, namely through the oral administration of free, bioidentical D-βHB. Results showed it to be well tolerated with only 6.2% of participants reporting any secondary symptoms. With the absence of a conjugate cation or intermediate metabolites, free D-βHB appears as a promising means of inducing sustained exogeneous ketosis although more research is needed to assess its clinical efficacy (82).

Although exogenous ketones raise the blood concentration to the levels we wish to obtain in the initiation and maintenance of a KD, caution should be applied when comparing the two distinctly separate metabolic states of endogenous and exogenous ketosis (66). Whilst exogenous ketones increase the circulating concentration of βHB, they are not in themselves ketogenic. Supplementation of exogenous ketones may rather inhibit ketogenesis in the liver (18). In spite of this, the rapidly elevated levels of βHB possible with exogenous ketones are of interest for the mitigation of some keto-induction side-effects when taken complimentary to a newly started KD (18). Kackley et al. monitored mood and cognitive performance during the initiation of a KD, both with supplementary ketone salts and with placebo. They observed lower depression scores and higher happiness levels among the ketone salt group, although the groups were not matched with regards to sodium intake and hence studies controlling for the increased cation load of the ketone salts are needed (27). In additional future studies, comparison of similar levels of ketosis induced endogenously or exogenously could help clarify to what extent ketones themselves might directly promote and/or alleviate different symptoms during keto-induction. Proposed dosing in the literature of ketone esters is 395–573 mg/kg body weight to achieve blood BOHB of 3.3 mmol/L. For a 80 kg male that would mean 31.6–45.8 grams (18). For ketone salts the proposed dose is 11.8 g (27). In both cases one would need to supplement regularly throughout the day to remain in ketosis since the baseline BHB concentration is reached again within 3–4 h (22).

##### 3.4.3.6 Kidney stone prophylaxis

During keto-induction there is increased urine excretion of calcium, phosphate and uric acid, and the urine becomes more acidic which predisposes individuals for precipitation of kidney stones. Potassium citrate can be used in conjunction with a KD as a prophylactic treatment for hypercalciuria and nephrolithiasis. Potassium citrate helps by alkalising the urine and thus increasing the solubility of free calcium. In a retrospective study of epileptic children receiving a KD at a single center, Wibisono et al. observed that after the introduction of potassium citrate as standard at their center none of the patients who were compliant with the supplementation programme developed kidney stones or related risk factors. The supplementation regime was a daily 2 mmol/kg/day for the duration of the KD, with this dose being adjusted and increased on the basis of spot urine measurements of calcium:creatinine >95 percentile or a citrate:creatinine < 0.3 mmol/mmol (29). In another study McNally et al. reported a 7-fold reduction in kidney stone incidence in patients receiving potassium citrate concurrently with a KD (29).

There are also recommendations in the literature to increase fluid intake to dilute the urine and thereby decrease the risk of precipitations and kidney stone formation. In a study of adults, a fixed volume of 3 L of water per day was suggested, but in most studies the recommendation is not quantified, but stated as “liberal fluid intake” (15, 73). In literature on non-ketogenic diets it has been shown that fluid intake >2 L/day significantly reduces the reoccurrence of kidney stones (83).

## 4 Discussion

Our literature review shows that transient symptoms associated with keto-induction are common, and that several mechanisms have been proposed for different symptoms. Reported common symptoms upon keto-induction include headache, light headedness, fatigue, lethargy, “brain fog,” decreased exercise capacity, mood changes, constipation, muscle cramps, diarrhea and halitosis. There are also reports of emesis, nausea, hypoglycaemia, acidosis, kidney stones and skin rash. The reviewed studies encompass a highly heterogenous population in terms of age, health status, inpatient and outpatient initiation, strictness of the diet, monitoring, method of initiation, formulation of the KD, food choices, and calorie restriction or *ab libitum* feeding. The studies also often use different nomenclature to denote similar symptoms, and use different methods to collect data on different sets of symptoms. Pediatric patients were often closely monitored at hospital during initiation, while adults were typically free living with intermittent follow-up. Consequently, the reported symptoms are highly variable in occurrence, and whether they are recorded varies between studies.

The identified studies have large variations in the formulations of KDs, from children on a formula-based KD to adults on a 4:1 fat: carbohydrate ratio diet, Atkins diet or ketogenic MCT-supplemented diet, and with varying content of whole foods, fiber and other food quality aspects. Some formulations are simply characterized as “well formulated KD” with non-starchy vegetables, nuts, seeds and minimally processed foods. Thus, some argue that many of the side-effects of keto-induction can be reduced if one adheres to a “well-formulated KD” (60), although there is no clear consensus what “well-formulated” specifically entails. The large variation in formulations of the studied KDs likely contributes to variations in the reported side-effects. There are also different methods of initiating the studied KDs. It appears that initiating a KD with fasting or gradual increase in calories is associated with more side-effect (32).

Despite these weaknesses of the available research, however, many of the same symptoms are reported across the different populations and diet formulations, supporting that there are some universal transient symptoms upon keto-induction. The most commonly reported symptoms of keto-induction are gastrointestinal symptoms such as obstipation for both children and adults. Nausea, diarrhea and vomiting are common for children, especially on KDs supplemented with MCT, which may at least partly be explained by delayed gastric emptying time and fat malabsorption that can occur with suddenly increased intake of fat. Light-headedness and dizziness are reported in adults, but not in children. For both adults and children, it is common to experience some form of reduced feelings of energy (fatigue, weakness, asthenia, lethargy). Halitosis is common in adults, but not documented in children. The occurrence of reduced exercise performance is not documented in any previous reviews but seems to be a common effect as it is reported in every study where it has been tested (although we cannot rule out publication bias, i.e., lack of publishing where no such symptoms have occurred). Muscle cramps are quite common for adults, but not for pediatric patients. Hypoglycaemia is only reported in the pediatric population, which could be because they are monitored more closely, or because of their reduced physiological reserves as compared to adults. The documentation is heterogenous for mood changes and “brain fog”/reduced cognitive performance. Less common but documented symptoms of keto-induction are acidosis and kidney stones in the pediatric population, whereas skin rash and prurigo pigmentosa are only reported in the adult population.

The proposed mechanisms of keto-induction are typically extrapolated forwards from known physiological changes in fasting and carbohydrate withdrawal, and backwards from the observed symptoms in trials of KD initiation. Natriuresis and kaliuresis is a well-documented phenomenon in the literature on both fasting and KD, although no studies have tied these states directly to symptoms of keto-induction. However, symptoms of mild hyponatremia, mild hypokalaemia and accompanying mild hypovolemia closely resemble observed symptoms of “keto-flu.” Another proposed mechanism of keto-induction is reduced energy substrate availability due to reduced serum glucose and low production of ketones within the first week. Again, this could explain many of the observed symptoms of reduced exercise performance, such as via fatigue, irritability, headache and dizziness. Yet, no studies have documented symptoms in relation to physiological findings. Moreover, it seems likely that keto-induction can increase the risk of kidney stones on account of both a physiological rationale with hypercalciuria, increased acid load and hyperuricemia and observed increased occurrence of kidney stones, especially in children.

Of note, although not directly relevant as symptoms during keto-induction, several of the studies report consistent changes in lipid metabolism for some individuals for both the pediatric and adult population. The state of ketosis can be associated with hypercholesterolemia in some individuals, with return to baseline following reintroduction of carbohydrates (84). For children some degree of both hypercholesterolemia and hypertriglyceridemia are reported, raising concerns about future cardiovascular disease (14, 15). In particular, a small increase in large density lipoprotein (LDL)-cholesterol is often observed, which might deter people from adopting—and physicians from advising—a KD. Some degree of hypercholesterolemia is also reported for adults. However, seemingly in contrast to children, adults show a consistent decrease in triglycerides, especially after an adaptation period (8, 11, 31, 60, 85–87). Thus, changes in any specific disease risk marker such as LDL cholesterol should be evaluated in the context of the overall metabolic changes, and the diet's overall therapeutic potential beyond any single disease. Importantly, KDs do not only typically decrease triglycerides (at least in adults), but also increase high-density lipoprotein (HDL) cholesterol and stabilize glucose-insulin physiology, which may contribute to many of the therapeutic benefits observed with KDs. Thus, the observed changes in cardiovascular risk profile following a KD are suggested to be net positive, regardless of a small increase in LDL cholesterol (31, 60, 85–87).

The proposed symptom mitigation interventions available in the literature are a mixed bag in terms of level of scientific rigor pointing in their favor. All the proposed strategies have a mechanistic approach that makes logical sense, but only a few of them have randomized controlled trials to back up the indication of their usage. Multiple strong studies showed that the traditional means of initiating a KD for inpatients, namely with a 24–48 h fast, is not more effective, whilst being associated with an increased burden of adverse symptoms, particularly in the youngest children, when compared to gradual initiation techniques and starting on a full calorie KD (26, 32, 64, 77).

While the use of MCT oil and exogenous ketones during diet initiation has a plausible physiological rationale for increasing energy availability before ketones fully replace glucose, the evidence supporting the clinical usefulness of their supplementation is limited and inconclusive. Supplementation of the KD with ketone salts showed encouraging improvements in mood scores, but there is considerable uncertainty regarding the positive contribution of the increased salt load supplied with the use of ketone salts, considering that the groups used were not matched for sodium intake. Therefore, it is not possible to conclude if the positive benefits from supplementing with ketone salt comes from increased energy availability or salt intake. Suggested dosing of ketone-salts in the studies are around 12 g every 3–4 h. On the other hand, MCT supplementation has been demonstrated to increase blood levels of βHB and improve mood scores and “symptom scores” for many symptoms of keto-induction. From the available literature supplementation with MCT oil in the form of 10–15 g of C8 and C10 three times per day appears to be a promising intervention to alleviate symptoms of keto-induction. However, MCT supplementation has its own dose-dependent side-effects with gastrointestinal distress, which requires gradual titration to be avoided. Ketone esters have only relatively recently become widely commercially available and thus little experimental evidence exists for their effects. However, they are the most potent aids of achieving ketosis, with the potential to induce serum ketone levels similar to starvation ketosis of 3–5 mmol/L. Thus, ketone esters may have the highest potential for alleviating symptoms related to reduced energy substrate availability during keto-induction since ketone esters are the most potent supplement for increasing blood ketones, and thereby increase available energy. But currently there is scarce evidence to support this hypothesis. Suggested dosing for ketone esters in the literature is 31–46 g every 3–4 h.

The natriuretic and kaliuretic effects of KD initiation are referred to in the vast majority of studies, but concrete studies and resulting quantified recommendations for salt supplementation during the KD initiation period do not appear to exist. Although Volek and Phinney have made a rough recommendation of an additional 1–2 g of sodium per day, this appears to be based on clinical experience and mechanistic rationale rather than empirical evidence. With respect to kidney stones, whilst strictly a longer-term complication of the KD than our study is focused upon, the prophylaxis of kidney stones with potassium citrate at a dosage of 2 mmol/kg/day appears to be a worthwhile intervention for children and at-risk adults. Studies have shown up to a 7-fold reduced risk of kidney stones with potassium citrate supplementation. Another intervention to prevent kidney stones is liberal fluid intake (>2 L/day), which has good documentation for non-ketogenic populations, and a robust mechanistic rationale for ketogenic kidney stone prophylaxis. Liberal fluid intake is also proposed as a treatment to compensate for the diuretic effect of natriuresis and kaliuresis during keto-induction and its proposed symptoms, however there is no experimental evidence to support this recommendation. Other proposed treatments to alleviate specific symptoms are adequate fiber intake to prevent/treat constipation, and controlled amounts of carbohydrates to counter hypoglycaemia. Increasing fiber intake can also be beneficial for the microbiome with possible implications for gastrointestinal and other symptoms. Recommended fiber intake in the literature is 15–20 g/day.

The aim of our systematic search was to retrieve the existing research on symptoms upon KD initiation, but we cannot rule out that relevant evidence has been missed. One significant pitfall is the sheer multitude of different terms used to refer to the initiation of a KD. It is possible that we have not accounted for a term or word within our search strategy that would have retrieved additional studies and added to the depth of our scoping review. Nonetheless, we are confident that we have reviewed a representative sample of the literature available. However, the ability to draw strong conclusions from the reviewed studies remains limited. Firstly, the heterogeneity of study designs with limited sample sizes and diverse study populations restricts the generalizability of the findings. Secondly, the reliance on self-reported symptoms can introduce bias and affect the reliability of the results. Thirdly, the review lacks studies that directly link reported symptoms during initiation with physiological measures such as electrolyte loss or microbiome changes. Similarly, many proposed interventions, such as MCT supplementation, are supported by limited studies, often without control groups, and require more empirical evidence to validate their effectiveness. The notable dearth of good quality randomized controlled trials on healthy adult populations in this field has meant that in several cases we have found ourselves drawing comparisons between studies based on adults with obesity eating *ad libitum* with studies on profoundly ill epileptic children being fed a controlled diet as inpatients. Despite many of the same symptoms, conclusions based upon epileptic children may well not be extrapolatable to other populations.

Several alternative approaches that seek to alleviate symptoms during keto-induction have been reviewed in the present work, which may help individuals and clinicians to optimize efficacy and tolerability and minimize potential adverse effects. The use of supplements such as MCT, electrolytes, amino acid like leucine and lysine and exogenous ketones have been suggested to aid in the induction of ketosis, and may possibly shorten the window and/or lessen the burden of symptoms. Some of these methods have proven effective in reducing the time required to achieve nutritional ketosis, and in some cases allowing for a greater proportion of dietary carbohydrate to be consumed whilst maintaining sufficient circulating ketone levels to remain in nutritional ketosis (18). With increasing evidence for the clinical usefulness of KDs, it should be important to fill the research gaps identified in the present scoping review, toward minimizing the common hurdles that may prevent adoption of a KD.

## Data Availability

The original contributions presented in the study are included in the article/supplementary material, further inquiries can be directed to the corresponding author.
